# Alcohol at bedtime induces minor changes in sleep stages and blood gases in stable chronic obstructive pulmonary disease

**DOI:** 10.1007/s11325-014-1020-y

**Published:** 2014-06-17

**Authors:** Nils Henrik Holmedahl, Britt Øverland, Ove Fondenes, Ivar Ellingsen, Jon Andrew Hardie

**Affiliations:** 1 LHL-klinikkene, Glittre, Glittreklinikken, postboks 104 Åneby, 1485 Hakadal, Norway; 2 ENT-Department, Lovisenberg Diakonale Hospital, Oslo, Norway; 3 Norwegian National Centre of Excellence in Home Mechanical Ventilation, Haukeland University Hospital, Bergen, Norway; 4 Department of Clinical Science, University of Bergen, Bergen, Norway

**Keywords:** Ethanol, Transcutaneous blood gas monitoring, Hypoventilation, COPD, Carbon dioxide, Polysomnography

## Abstract

**Purpose/background:**

The purpose of this study is to explore the effect of a moderate dose of alcohol on sleep architecture and respiration in chronic obstructive pulmonary disease (COPD). Alcohol depresses both hypercapnic and hypoxic ventilatory drives in awake, normal individuals and reduces the amount of rapid eye movement (REM) sleep and oxygen saturation (S_p_O_2_) in sleeping COPD subjects.

**Methods:**

Prospectively designed, open-label interventional study in a pulmonary rehabilitation hospital. Twenty-six (nine males) stable inpatients, median forced expiratory volume first second (FEV1) 40.5 % of predicted, median age 65 years, investigated by polysomnography including transcutaneous measurement of carbon dioxide pressure increase (ΔP_tc_CO_2_) in randomized order of either control sleep or intervention with 0.5 g of ethanol/kilogram bodyweight, taken orally immediately before lights off.

**Results:**

Alcohol induced a mean increase (95 % confidence interval, [CI]) in the mean ΔP_tc_CO_2_ of 0.10 kPa (0.002–0.206, *P* = 0.047) and a mean decrease (CI) in the REM-sleep percentage of total sleep time (REM % of TST) of 3.1 % (0.2–6.0), (*P* = 0.020). Six subjects with apnea/hypopnea index (AHI) ≥15 had *fewer* apneas/hypopneas during alcohol versus control sleep (mean reduction of AHI 11 (1–20), *P* = 0.046). Alcohol-sleep changes in S_p_O_2_, but not in ΔP_tc_CO_2_, correlated with daytime arterial pressures of carbon dioxide (P_a_CO_2_) and oxygen (P_a_O_2_).

**Conclusion:**

Occasional use of a moderate, bedtime dose of alcohol has only minor respiratory depressant effects on the majority of COPD subjects, and in a minority even slightly improves respiration during sleep. However, caution is appropriate as this study is small and higher doses of alcohol may result in major respiratory depressive and additional negative health effects.

## Introduction

Chronic obstructive pulmonary disease (COPD) has a significant morbidity and mortality [1], and chronic hypercapnic respiratory failure (CHRF) in COPD is associated with poor prognosis [2, 3]. Heavy alcohol consumption has been shown to increase the risk of developing COPD [4–6]; on the other hand, mild alcohol intake may reduce the risk both of developing severe pulmonary function abnormalities and of dying from COPD [7, 8]. Sleep is characterized by periods of unstable respiration, and in normal, awake individuals, hypoxic and hypercapnic ventilatory drives are depressed by alcohol [9]. Sleep hypoventilation (SH) has previously been shown to be frequent in severe COPD with CHRF and long-term oxygen therapy (LTOT) [10–12], and recently, we also found SH in some normocapnic, non-LTOT individuals with stable COPD [12]. Both in a study of 20 asymptomatic men aged 20 to 65 years [13] and in a study of 20 COPD subjects (19 men) [14], alcohol increased the number of sleep apneas. However, a study of 18 asymptomatic, postmenopausal women found no difference between placebo- and alcohol-influenced sleep in the number of episodes of apnea or hypopnea or in the frequency, length, or severity of oxygen desaturation [15]. In two small studies of four normal individuals and five subjects with stable COPD, alcohol taken immediately before sleep resulted in a moderately reduced mean S_p_O_2_ and a reduced amount of rapid eye movement (REM) sleep [16, 17]. To our knowledge, no studies have explored alcohol-induced changes in carbon dioxide pressure (PCO_2_) with polysomnography (PSG) in sleeping COPD patients. Concern is thus raised whether it is safe for individuals with COPD, especially those with CHRF or frequent apneas/hypopneas, to occasionally consume alcohol before sleep.

Hypothesizing an increase in the PCO_2_ during sleep, the primary aim of this study was to explore to what extent a moderate dose of alcohol prior to sleep induces hypoventilation in stable COPD patients, secondary whether apneas/hypopneas and sleep architecture is altered, and finally whether the alcohol-induced changes in blood gases during sleep is correlated to daytime COPD characteristics.

## Material and methods

### Subjects

**Fig. 1:**
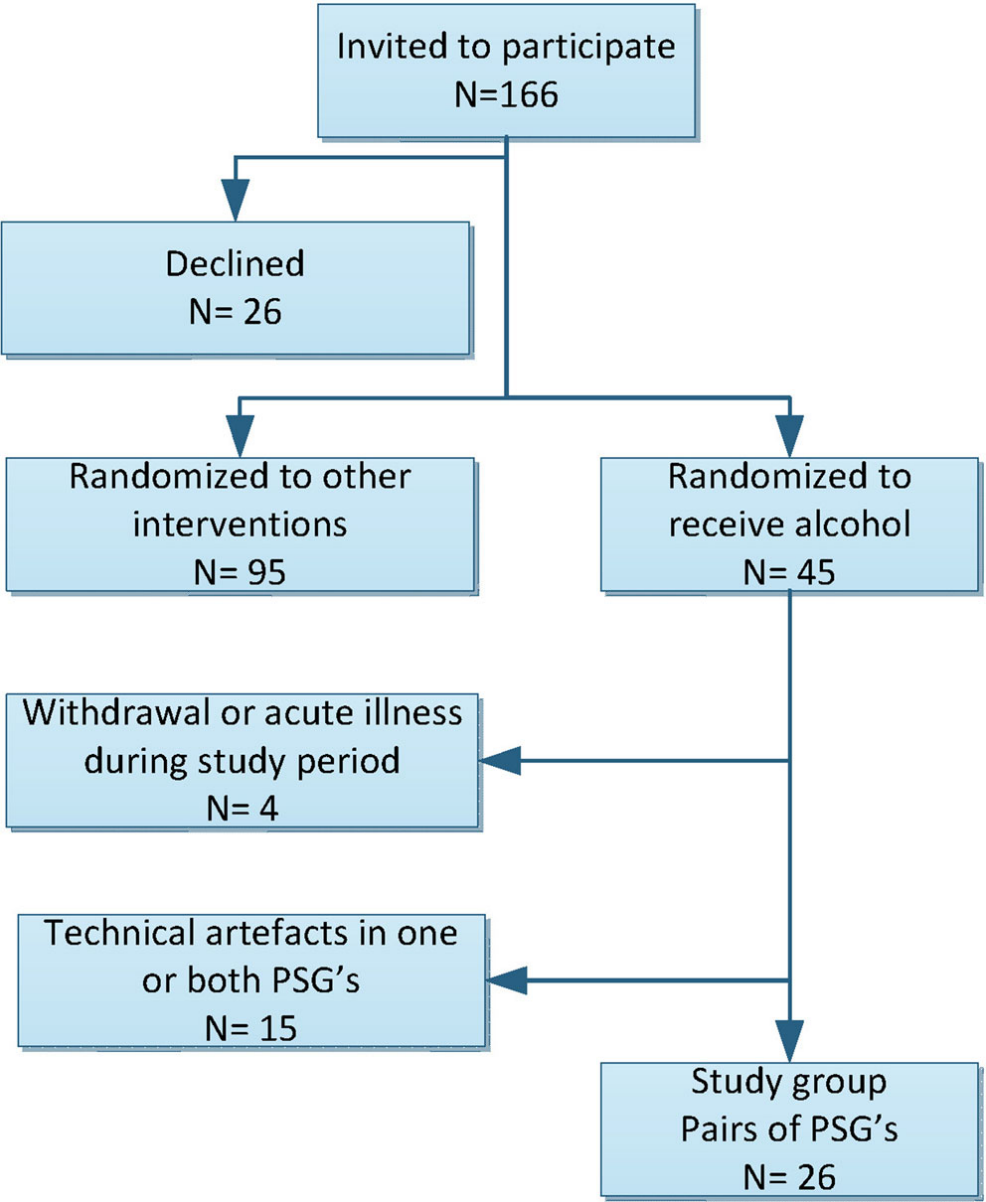
Inclusion

Study participants were a randomized selection from the 166 subjects described in a previous article [12], all Caucasians with GOLD [18] defined COPD and inpatients at the Glittreklinikken Pulmonary Rehabilitation Hospital included from January 2010 through June 2011. The following exclusion criteria were used: diagnosed obstructive sleep apnea (OSA); COPD exacerbation within 3 weeks prior; other serious lung comorbidity (i.e., cancer, sarcoidosis, restrictive lung disease) or diseases affecting thoracic or abdominal movement, unstable angina pectoris, hypertension, diabetes mellitus, myocardial infarction within last 3 months, cerebral infarction, and addiction to drugs, alcohol, or narcotics. At inclusion, study subjects were randomized to receive alcohol, 5 mg of Zopiclone (a sleep drug), or supplementary oxygen (data from the latter two interventions not yet published). All subjects used prescribed medication, but no drugs known to be respiratory depressants were taken from 48 h prior to first PSG recording until end of study. The protocol was approved by the Regional Ethics Committee in south-eastern Norway. As shown in Fig. 1, 26 pairs of PSG from control and alcohol-influenced sleep were analyzed.

### Measurements

All subjects slept in their own bed at the hospital with full PSG for three nights; first night for acquaintance with the equipment, second and third nights randomized to either control or alcohol-influenced sleep. PSG was recorded online from Embla A10^1^ with transcutaneously measured carbon dioxide pressure (P_tc_CO_2_) from Tosca 500^2^ to a bedside computer using Somnologica Studio Version 3.3 software. Channel setup was according to the 2007 recommendations from the American Academy of Sleep Medicine (AASM) [19]. PSGs were recorded only during weekdays. Alcohol 0.5 mg per kg bodyweight (corresponding to about three glasses of wine in a 70-kg person) was given as 96 vol% ethanol diluted in 200 ml of orange juice and ingested the last 30 min prior to sleep [20]. Immediately before lights off, breath analysis by Lion alcometer 500^3^ was used to check that study-alcohol was taken.

Unpublished data collected at Glittreklinikken prior to the study showed equivalence between changes in P_tc_CO_2_ and P_a_CO_2_, but a delay time of 54–57 s. Details regarding delay time, P_tc_CO_2_ signal stabilization, sampling frequency, and calculations according to sleep stages are given in our previous article [12].

Arterial blood gas samples^4^ were collected after a 10-min seated rest at approximately 2 p.m. prior to each PSG recording and analyzed within 10 min on the Radiometer ABL720Flex.^5^ At sampling, all subjects were breathing room air, with an exception for those on LTOT who used their prescribed dose of supplementary oxygen. Height, weight, and lung function tests including postbronchodilator spirometry, diffusing capacity (DLCO), and body plesmography of total lung volumes were recorded on MasterScreen Pneumo^6^ as previously described [12], reference values are based on equations from the European Community for Coal and Steel [21].

Sleep scoring was done by two experienced polysomnographists according to recommendations from the AASM [12, 19]; a hypopnea was scored when nasal pressure dropped ≥30 % for ≥10 s with ≥4 % desaturation drop from baseline, with ≥90 % of the event’s duration meeting the amplitude reduction criteria for hypopnea (criterion A). Sleep hypoventilation is defined by the AASM as an increase of 1.3 kPa or more in P_a_CO_2_ during sleep, to a value exceeding 6.7 kPa for at least 10 min [22].

### Statistical analysis

Sample size calculations up front showed that for *N* = 28, a P_tc_CO_2_ difference between alcohol and control sleep would be detected with 80 % probability at a two-sided 0.05 significance level if the true difference was 0.25 kPa, assuming a within-patient standard deviation of 0.32 kPa^7^

Data were assessed for normality of distribution and homogeneity of variance. No violation of the assumptions of normality, linearity, multicolinearity, and homoscedasticity was found preliminary to regression analysis.

Differences between groups with continuous, paired parametric data were analyzed with Student *T*, paired non-parametric data with Wilcoxon signed rank, independent non-parametric data with Mann-Whitney *U*, and non-parametric data in more than two groups by Kruskal-Wallis tests. Paired proportions were analyzed with McNemar chi-square test (*P* value uncorrected) and correlations with Pearson’s *r*.

Two-sided *P* values of ≤0.05 were considered significant in testing the main hypothesis, whereas a significance level of *P* ≤ 0.025 was applied in the remaining analyses. All analyses were performed using IBM SPSS Statistics version 19.

## Results

The 9 men and 17 female (Table 1) were on average hyper-inflated with impaired gas exchange and severe airway obstruction, 14 (54 %) of the 26 subjects at GOLD stages III and IV. Four subjects (16 %) had daytime hypoxemia (arterial pressures of oxygen (P_a_O_2_) < 8.0 kPa), whereas only one had P_a_O_2_ > 12.0 kPa. CHRF (P_a_CO_2_ ≥ 6.3 kPa) was found in four subjects, of which three used LTOT. The study group had a normal median BMI, five subjects (19 %) being underweight (BMI < 21), whereas five were obese (BMI ≥ 30).

**Table 1:** Study population

	*N* = *26* Median (IQR)
Demographic data	
Gender female	17 (65)^a^
Age, years	65.5 (14.0)
BMI, kg/m^2^	25.0 (6.5)
Smoking habit	
Pack years	30.4 (22.4)
Current smoker	4 (15)^a^
Medication	
LTOT	3 (12)^a^
SABA/LABA	24 (92)^a^
Statin	4 (15)^a^
ACE/AII	3 (12)^a^
Thiazide	3 (12)^a^
ASA	3 (12)^a^
Spirometry	
FVC, % of pred	81.0 (35.3)
FEV1, % pred	40.5 (30.3)
FEV1/FVC, ratio	0.46 (0.21)
DLCO^1^, mmol/min/kPa	3.92 (2.49)
RV/TLC^2^, ratio	0.56 (0.20)
Clinical data	
CAT, score	18 (7)
MMRC, score	2.0 (1.0)
6-MWD^3^, meter	420 (205)
BODE index	2.0 (4.0)
Laboratory data	
P_a_O_2_, kPa	9.66 (1.98)
P_a_CO_2_, kPa	5.07 (.96)
S_a_O_2_, %	96.2 (3.3)
Alcohol, ppt	0.81 (0.64)

*IQR* inter quartile range, *BMI* body mass index, *LTOT* long-term oxygen therapy, *SABA/LABA* short- and/or long-acting beta-2 receptor agonist, *ACE/AII* angiotensin converting enzyme and/or angiotensin II antagonist, *Thiazide* hydrochlorothiazide, *ASA* acetylic salicylic acid, *FVC % of pred* forced vital capacity as percent of predicted value, *FEV1% of pred* forced expiratory volume first second as percent of predicted value, *DLCO* diffusing capacity of the lung for carbon monoxide, *RV* residual volume, *TLC* total lung capacity, *CAT* COPD assessment test, *MMRC* modified medical research council questionnaire, 6-*MWD* 6 min walking distance, *BODE* BMI/obstruction/dyspnea/exercise capacity, *P*_*a*_ *O*_*2*_ arterial pressure of oxygen, *P*_*a*_ *CO*_*2*_ arterial pressure of carbon dioxide, *S*_*a*_ *O*_*2*_ arterial oxygen saturation,. *Alcohol* parts per thousand measured by breath analysis immediately prior to sleep

^a^ *N* (% of study subjects), instead of median (IQR)

^1^ *N* = 22, four missing from DLCO because of insufficient vital capacity or because they could not hold their breath for 10 s

^2^ *N* = 24, two missing from body plethysmography because of claustrophobia

^3^ *N* = 25, one missing from 6-MWD, reason unknown

**Fig. 2:**
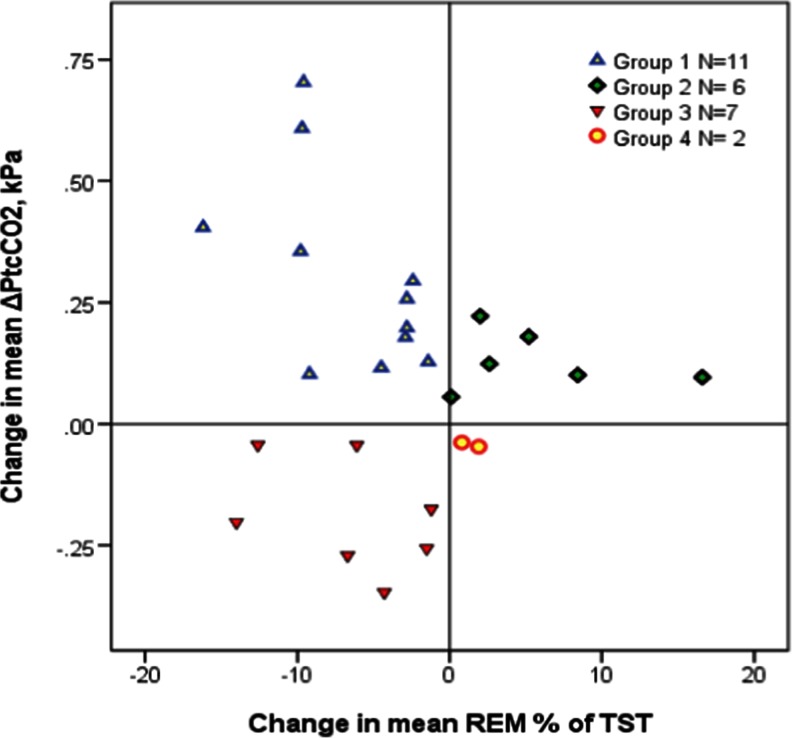
Alcohol-induced changes in mean ΔP_tc_CO_2_ versus changes in REM-sleep percentage of TST. Each subject (*N* = 26) is represented by a colored figure according to group (legend in panel). Mean P_tc_CO_2_ in group 1 (median (IQR) 0.26 (0.28) kPa) differed significantly from group 3 (−0.20 (0.23) kPa, *P* < 0.0005), but not from group 2 (0.11(0.10) kPa, *P* = 0.027). The change in REM percentage of TST was significantly different between groups 1 and 2 (median (IQR) = −4.5 (6.9) % versus 3.9 (8.9) %, *P* = 0.001). *Change in mean ΔP*_*tc*_ *CO*_*2*_ is the difference in the mean increase in transcutaneous carbon dioxide pressure between alcohol and control sleep. *Change in mean REM % of TST* is the difference in the mean rapid eye movement sleep percent of total sleep time between alcohol and control sleep

As indicated in Table 2, alcohol at bedtime reduced the REM-sleep percentage of total sleep time (REM % of TST) with a mean (SD) of 3 (7) %, and the number of awakenings with 8 (13). However, eight subjects had an *increased* amount of REM sleep when influenced by alcohol (Fig. 2, groups 2 and 4).

**Table 2:** Control versus alcohol-influenced sleep

*N* = 26	Control sleep Median (IQR)	Alcohol sleep Median (IQR)	*P* value
Sleep architecture			
TST, min	354.0 (65.3)	354.0 (62.5)	0.501
REM % of TST	25 (8)	20 (9)	0.020
NREM % of TST	75 (8)	80 (9)	0.020
WASO, min	45.7 (74.7)	40.9 (48.5)	0.361
Awakenings	28 (18)	19 (17)	0.012
AI	15.5 (10.0)	18.6 (10.0)	0.920
Ventilation			
AHI	9.7 (10.2)	10.4 (16.4)	0.517
HI	4.8 (6.2)	6.3 (8.6)	0.309
ODI^a^	5.5 (11.6)	8.3 (12.8)	0.548
Mean S_p_O_2_ %			
N0	94.0 (5.1)	93.9 (5.6)	0.551
NREM	93.7 (4.6)	93.2 (5.4)	0.019
REM	92.5 (5.4)	92.8 (6.4)	0.258
Sleep	93.4 (4.7)	93.2 (5.7)	0.058
Mean ΔP_tc_CO_2_ kPa			
N0	0.19 (0.23)	0.15 (0.22)	0.638
NREM	0.39 (0.34)	0.55 (0.36)	0.035
REM	0.67 (0.46)	0.67 (0.42)	0.082
Sleep	0.49 (0.35)	0.58 (0.36)	0.047
Sleep hypoventilation			
SH^b^	8 (27)	19 (40)	0.179

*TST* total sleep time, *REM* rapid eye movement sleep, *NREM* non-REM sleep, *WASO* wake after sleep onset, *Awakenings* number of awakenings after sleep onset, *AI* number of arousals per hour (arousal index), *AHI* number of apneas/hypopneas per hour (apnea/hypopnea index), *HI* number of hypopneas per hour (hypopnea index), *ODI* number of oxygen desaturations per hour (oxygen desaturation index), *S*_*p*_ *O*_*2*_ oxyhemoglobin saturation by pulse oximetry, *N*0 awake after initial sleep onset, *Sleep* = NREM + REM, *ΔP*_*tc*_ *CO*_*2*_ carbon dioxide pressure increase from supine, resting value prior to sleep, *SH* sleep hypoventilation defined by the American Academy of Sleep Medicine as an increase of 1.3 kPa or more in P_a_CO_2_ during sleep, to a value exceeding 6.7 kPa for at least 10 min

^a^ *N* = 25, one missing because of defect finger probe

^b^Percent of “Yes” within group (SD), instead of median (IQR)s

The mean P_tc_CO_2_ increase from supine, resting value prior to sleep (ΔP_tc_CO_2_) was higher during alcohol versus control sleep (a mean (SD) increase of 0.10 (0.25) kPa), despite nine subjects having a *decrease* in the mean ΔP_tc_CO_2_ (Fig. 2, groups 3 and 4). As shown in Table 4, alcohol-sleep changes in mean ΔP_tc_CO_2_ and mean S_p_O_2_ did not correlate.

Sleep hypoventilation (SH) as defined by the AASM was found in two subjects (8 %) during control sleep and in five subjects (19 %) in alcohol sleep [22]; however, the difference was not statistically significant as one subject with SH in control sleep had no SH during alcohol sleep.

Ten subjects had a drop in alcohol-sleep mean S_p_O_2_ of >1 %, whereas the NREM sleep mean S_p_O_2_ for the whole study group decreased with a mean (SD) of 0.9 (2.1) % (*P* = .019). No significant change was found in REM sleep (Table 2). Interestingly, Table 2 also shows no respiratory depressant effect of alcohol during the awake periods after the initial sleep onset (N0) nor was there significant N0 changes in mean ΔP_tc_CO_2_ and mean S_p_O_2_ when analyzing only subjects with alcohol increased sleep mean ΔP_tc_CO_2_ (groups 1 and 2, Fig. 2).

The median values of apneas/hypopneas and desaturations per hour of sleep (AHI and ODI, respectively) were not changed by alcohol. Despite the exclusion of diagnosed OSA prior to the study, six subjects (four males) had an AHI of ≥15 per hour in control sleep. Intriguingly, Table 3 indicates that this subgroup had alcohol sleep *decreases* in median AHI and ODI, all with increased mean ΔP_tc_CO_2_ and decreased REM % of TST.

**Table 3:** Alcohol-induced sleep changes in overlap subjects

*N* = 6 (four male)	Control sleep Median (IQR)	Alcohol sleep Median (IQR)	*P* value
REM % of TST	25 (12)	21 (15)	0.028
NREM mean ΔP_tc_CO_2_, kPa	0.48 (0.39)	0.68 (0.27)	0.028
REM mean ΔP_tc_CO_2_, kPa	0.64 (0.63)	0.91 (0.35)	0.028
Sleep mean ΔP_tc_CO_2_, kPa	0.51 (0.46)	0.75 (0.28)	0.028
Sleep mean S_p_O_2_, %	93.3 (3.8)	93.2 (6.5)	0.075
ODI	32 (28)	24 (22)	0.028
AHI	39 (40)	26 (22)	0.046
REM AHI	49 (43)	36 (28)	0.075
NREM AHI	31 (46)	30 (23)	0.116

As no record of daytime sleepiness was available, overlap was defined as having at least 15 apneas/hypopneas per hour of sleep in subjects with chronic obstructive pulmonary disease

*NREM mean ΔP*_*tc*_ *CO*_*2*_ mean pressure of transcutaneous carbon dioxide in non-rapid eye movement sleep, *REM mean ΔP*_*tc*_ *CO*_*2*_ mean pressure of transcutaneous carbon dioxide in rapid eye movement sleep, *Sleep mean ΔP*_*tc*_ *CO*_*2*_ mean pressure of transcutaneous carbon dioxide in REM and NREM sleep, *Sleep mean S*_*p*_ *O*_*2*_ mean oxyhemoglobin saturation during sleep measured by pulse oximetry, *REM % of TST* REM-sleep percentage of total sleep time, *AHI* number of apneas/hypopneas per hour of sleep, *REM AHI* AHI during REM sleep, *NREM AHI* AHI during NREM sleep

As the three LTOT users were outliers in the data distribution of the mean S_p_O_2_ change, partial correlation was applied, controlling for LTOT use (yes/no). Alcohol-sleep changes in the mean S_p_O_2_ (∆_AS_S_p_O_2_) were highly correlated to daytime P_a_O_2_ and inversely to P_a_CO_2_ (Table 4). However, this was not the case regarding the alcohol-sleep changes in the mean ΔP_tc_CO_2_ nor did hypoventilation during alcohol sleep correlate to spirometry or demographic data.

**Table 4:** Correlation between alcohol-induced blood gas changes in sleep and study population characteristics

*N* = 26	Change in mean S_p_O_2_^a^		Change in mean ΔP_tc_CO_2_	
	Pearson’s *r*	*P* value	Pearson’s *r*	*P* value
Demographic data				
Age, years	−0.11	0.616	0.02	0.914
Gender	0.08	0.709	0.06	0.784
BMI kg/m^2^	−0.13	0.543	0.13	0.516
Laboratory data				
P_a_O_2_, kPa	0.51	0.009	−0.25	0.227
P_a_CO_2_, kPa	−0.58	0.002	0.11	0.604
pH	0.37	0.071	0.05	0.816
Spirometry				
FVC % of pred	0.28	0.170	−0.04	0.855
FEV1 % of pred	0.32	0.125	−0.03	0.886
DLCO mmol/min/kPa^b^	0.32	0.161	−0.23	0.308
RV/TLC ratio^c^	−0.28	0.202	0.01	0.950
Sleep				
Change in REM % of TST	0.12	0.554	−0.15	0.454
Change in awakenings	−0.11	0.602	0.22	0.274
Change in mean S_p_O_2_	−	−	−0.30	0.135

*Change in mean S*_*p*_ *O*_*2*_ difference between alcohol and control sleep in the mean oxygen saturation, *Change in mean ΔP*_*tc*_ *CO*_*2*_ difference between alcohol and control sleep in the mean increase from awake, supine, transcutaneous carbon dioxide pressure, *BMI* body mass index, *P*_*a*_ *O*_*2*_ arterial pressure of oxygen, *P*_*a*_ *CO*_*2*_ arterial pressure of carbon dioxide, *FVC % of pred* forced vital capacity as percent of predicted value, *FEV1% of pred* forced expiratory volume first second as percent of predicted value, *DLCO* diffusing capacity of the lung for carbon monoxide, *RV* residual volume, *TLC* total lung capacity, *Change in REM % of TST* difference in the mean rapid eye movement sleep percent of total sleep time between alcohol and control sleep, *Change in awakenings* difference in the number of awakenings between alcohol and control sleep

^a^Partial correlation controlling for LTOT use is tabulated

^b^Four missing from DLCO because of insufficient vital capacity or because they could not hold their breath for 10 s

^c^Two missing from body plethysmography because of claustrophobia

Finally, hierarchical multiple regressions were performed to determine whether the ∆_AS_S_p_O_2_ could be explained by daytime COPD characteristics. P_a_O_2_ and P_a_CO_2_ were entered at step 1, explaining 34 % of the variance in ∆_AS_S_p_O_2_. After the entry of LTOT (yes/no) at step 2, the total variance explained by the model was 49 %, (*F*(3, 22) = 7.09, *P* = 0.002). Thus, LTOT (yes/no) explained an additional 15 % of the variance in the ∆_AS_S_p_O_2_ after controlling for P_a_O_2_ and P_a_CO_2_ (*R* ^2^ change = 0.015, *F* change (1, 22) = 6.48, *P* = 0.018). In the full model, using LTOT recorded the highest beta value (0.70, *P* < .018), over P_a_CO_2_ (−0.59, *P* < 0.031), whereas P_a_O_2_ did not make a unique contribution (0.31, *P* = 0.116).

## Discussion

When stable COPD subjects (2/3 female) are given a moderate dose of alcohol immediately prior to sleep, we find a very modest sleep hypoventilation as a group mean increase in P_tc_CO_2_ of about 0.1 kPa and a decrease in S_p_O_2_ of less than 1 %. This hypoventilation is not detectable when subjects are awake after the initial sleep onset. The mean REM % of TST is decreased in alcohol sleep. A novel finding is subgroups showing opposite characteristics as slight hyperventilation with decreased amount of REM sleep and hypoventilation with increased REM percentage of TST. AHI and ODI are not significantly increased by alcohol; however, in overlap subjects (AHI ≥15/h), AHI and ODI are lower. Alcohol-sleep change in S_p_O_2_, but not P_tc_CO_2_, is associated to daytime blood gases, and a model with LTOT, P_a_CO_2_, and P_a_O_2_ can explain about half of the variation in alcohol sleep change in S_p_O_2_.

In a much referred study from 1967, Yules et al. gave 1 g of ethanol/kg body weight to four male graduate students 4 h prior to sleep and found that the REM-sleep time reduced as a mean for the group; however, one subject showed no change in REM-sleep time [16]. Later, Easton et al. gave five COPD subjects 0.78 g of alcohol/kg bodyweight immediately prior to sleep and found a mean TST decrease of 19 %, a REM-sleep percentage of TST decrease by 12 % and a mean drop in S_p_O_2_ of about 3 % [17]. In 2007, Brander et al. studied nine males with advanced COPD using 0.5 g of alcohol/kg bodyweight albeit taken earlier in the evening, they did not record PSG but found only a trivial decrease in S_p_O_2_ (<1 %) during the first 2 h of the reported sleep [23].

In the present study, we find REM % of TST decreased as in the studies referred above, however, only in 18 (69 %) of the subjects. One possible explanation for the eight subjects showing *increased* REM % of TST can be alcohol abstinence. Easton et al. found the REM % of TST gradually increasing when giving alcohol repeatedly for several nights. Although none of our study subjects had a drinking problem (otherwise they would not have been included), some could nevertheless have used alcohol regularly before admission, and thus the abstinent state during the first week at the hospital might explain the increase in REM sleep when given alcohol. However, we find it unlikely that as many as 31 % of the study subjects had an undisclosed alcohol problem, and as Yules et al. also described one subject with no change in REM-sleep time [16], hitherto unknown effects of alcohol on sleep can have caused the increased mean REM % of TST in some of the subjects in the present study (Fig. 2).

In a previous article, we showed that ΔP_tc_CO_2_ increases with depth of NREM sleep in stable COPD and with the highest values recorded during REM sleep [12]. Thus, finding the REM percentage of TST being *reduced* in alcohol sleep, one would expect to find the mean ΔP_tc_CO_2_ to be *decreased*. Indeed, although not clinically impressive, the seven (27 %) subjects in group 3 in Fig. 2 fit in this picture. Brief alcohol exposure is moderately bronco-dilating and increases the mucociliary motility in asthmatics [24], and thus can relieve nocturnal wheezing in reversible COPD subjects, and possibly improve gas exchange in the lungs. However, the 11 (42 %) subjects with reduced REM % of TST and *increased* P_tc_CO_2_ (group 1, Fig. 2) are more in accordance with previous findings in COPD [17]. The hypoventilation demonstrated in the present study with only two subjects (8 %) having a mean alcohol-sleep increase in ΔP_tc_CO_2_ > 0.5 kPa and less than half having a drop in mean SpO2 > 1 % was considerably less impressive than Easton et al.’s findings and more in line with the results of Brander et al.[17, 23]. One obvious explanation is the amount of alcohol and the time of ingestion related to sleep onset, as Brander et al. gave less alcohol earlier in the evening compared to Easton et al., the present study using same amount of alcohol as Brander et al., but given immediately before lights off. Thus, as REM sleep is more frequent the second half of the night when most of the alcohol is metabolized, REM-sleep hypoventilation is not significantly changed. Another explanation can be the number of study subjects; as previous studies included only up to six subjects, they could have missed the individuals having decreased P_tc_CO_2_ during alcohol sleep.

Previous studies have shown increased numbers of apneas in alcohol-sleep in asymptomatic and in snoring males, in mild to moderate OSA-males and in male, but not in female COPD subjects [13, 25, 26, 14, 15]. The present study did not find AHI, HI, and ODI significantly increased by alcohol. This can partly be explained by the majority of female subjects in the present study. Also, as apneas/hypopneas are most frequent in REM sleep, occurring at low alcohol content, the reduced REM % of TST in alcohol-sleep can result in the total AHI being unchanged. However, the subgroup of mostly men with control sleep AHI ≥ 15/h having *decreased* AHI and ODI during alcohol sleep seems to contradict previous findings in males. All these individuals are hypoventilating, yet they have fewer apneas, and although not statistically significant, the REM-sleep AHI tends to be lower in alcohol-sleep. A reduced tidal volume with increased frequency of respiration, shorter episodes of REM-sleep, less mucus, and reduced bronchial constriction can be possible explanations.

Interestingly, no changes are found in ΔP_tc_CO_2_ or S_p_O_2_ in N0 (awake after initial sleep onset), suggesting that moderate doses of alcohol has a significant effect on the central respiratory control only during sleep. This is in support of Sahn et al., who did not find significant alcohol-induced hypoventilation (neither as decreased PO_2_ nor increased PCO_2_) in six awake subjects with severe COPD after drinking 0.78 g of alcohol/kg bodyweight [27].

Having COPD with daytime respiratory failure correlates with alcohol-induced sleep hypoventilation, however, only as daytime blood gases versus ∆_AS_S_p_O_2_, and not hypoventilation as change in mean ΔP_tc_CO_2_ (Table 4). As the multiple linear regressions with the variables LTOT, P_a_O_2_, and P_a_CO_2_ can explain only half of the decrease in S_p_O_2_ in alcohol sleep, advice to COPD patients regarding the use of alcohol cannot be based on whether they have respiratory failure or not. Nor is the severity of COPD according to FEV1 as percentage of predicted, the transfer factor for carbon monoxide (DLCO) or the residual volume as percentage of total lung volume (RV/TLC) highly associated with the alcohol-induced sleep hypoventilation.

This study is to our knowledge the first to explore sleep hypoventilation as both oxygen desaturation and carbon dioxide increase in moderately alcohol-intoxicated COPD subjects; however, it has some limitations. Many comparisons increase the risk of type 1 statistical errors (falsely rejecting the null hypothesis when in fact it is true) and although an alpha-correction was applied in interpreting the results of analyses other than the main hypothesis, the significance of the results must be viewed with this risk in mind. The open-label design can add a placebo bias to the alcohol intervention. However, we find it less likely that ventilation during sleep is affected by the knowledge of being drunk, and the fact that blood gases were unchanged when awake after initial sleep onset indicates little placebo impact. Another limitation is the lack of a detailed, structured alcohol consumption interview, as regular alcohol ingestion, although not being considered as a drinking problem, can result in a very different sleep and breathing patterns as discussed above. Thirdly, no records were made of whether or not the study subjects were fasting at the time of alcohol ingestion. The alcohol breath test performed immediately before lights off was not considered a valid measurement of blood alcohol content (BAC) as such analyses are considered to be unreliable in COPD [28–30]; nevertheless, the breath analyses showed an unexpected high variability (Table 1). Food taken together with alcohol can delay peak BAC, possibly explaining some of this variability. If BAC correlates with ΔP_tc_CO_2_, fasting individuals can have peak BACs and thus high ΔP_tc_CO_2_, values early in the night, compared to those with their BAC peak well into the night. However, supper at the hospital was at 6:30 p.m., and we find it unlikely that many study subjects ate at a later time.

## Conclusion/summary

In this study of stable COPD patients drinking a moderate dose of alcohol immediately prior to lights off, we found a very modest sleep hypoventilation as increased mean ΔP_tc_CO_2_, a reduced REM % of TST, and no changes in AHI. However, this combination of hypoventilation and decreased REM-sleep time was evident in less than half of the subjects, as about one-fourth had a minor sleep *hyperventilation* with reduced REM % of TST and another one-fourth was hypoventilating with an *increase* in REM % of TST. Also, subjects with COPD and OSA (overlap) had *lower* median AHI and ODI in alcohol-sleep versus control sleep. We found an association between daytime blood gases and alcohol-sleep hypoventilation as decreased S_p_O_2_, but not increased ΔP_tc_CO_2_, and a regression model with the variables LTOT-use, P_a_O_2_, and P_a_CO_2_ could explain 49 % of the variation in alcohol sleep S_p_O_2_ change. As the blood gas changes were minor, the clinical implication of this study is that a single, moderate, bedtime dose of alcohol seems to have only minor respiratory depressant effects on the majority of COPD subjects, and in a minority even slightly improves respiration during sleep. However, caution must be applied as the study is small, and higher doses of alcohol may result in major respiratory depressive and additional negative health effects.

## Footnotes

1 Medcare Flaga, Reykjavik, Iceland.

2 Radiometer, Basel, Switzerland.

3 Lion Laboratories Ltd., Vale of Glamorgan, UK.

4 PICO50, Radiometer, Copenhagen, Denmark.

5 Radiometer, Copenhagen, Denmark.

6 Jaeger-Toennies, Hoechberg, Germany.

7 In our previous study of 100 COPD subjects [12], the mean increase (standard deviation) in P_tc_CO_2_ during spontaneous sleep was 0.49 (0.32) kPa.
